# Tacorin, an extract from *Ananas comosus* stem, stimulates wound healing by modulating the expression of tumor necrosis factor α, transforming growth factor β and matrix metalloproteinase 2

**DOI:** 10.1002/2211-5463.12241

**Published:** 2017-06-09

**Authors:** Puji Rahayu, Lia Agustina, Raymond R. Tjandrawinata

**Affiliations:** ^1^ Biopharmaceutical Technology DivisionDexa Laboratories of Biomolecular SciencesPT Dexa MedicaCikarangIndonesia

**Keywords:** *Ananas comosus* stem, Tacorin, wound healing accelerator

## Abstract

Wound healing is a complex biological process that involves integration of hemostasis, inflammation, proliferation and tissue remodeling. An extract of pineapple (*Ananas comosus*) stem demonstrates several therapeutics properties, including acceleration of wound healing. Tacorin is a water crude extract derived from the stem of *A. comosus* with high protein content. The effect of tacorin on wound healing *in vivo* was examined using rats with an induced injury. Wound closure was faster with tacorin treatment than in the untreated group. An *in vitro* study was conducted on mammalian cells (3T3‐L1) to observe the effect of tacorin on cell proliferation. Tacorin was first heated to inactivate its proteolytic activity. It increased the viability of 3T3‐L1 cells in a dose‐dependent manner. Excessive inflammation was suppressed by tacorin as shown by decreased tumor necrosis factor α expression. Treatment with tacorin increased the expression of transforming growth factor β, a major player in tissue remodeling. Moreover, tacorin also reduced the expression of MMP‐2 to accelerate the recovery of the wound. Taken together, tacorin is able to accelerate the wound‐healing process by increasing cell proliferation, suppressing inflammation and accelerating tissue remodeling.

**Abbreviations**
DLBS: Dexa Laboratories of Biomolecular Sciences
IL: interleukin
MMP: matrix metalloproteinase
TGF: transforming growth factor
TNF: tumor necrosis factor

A complex series of interactions between different cell types, cytokine mediators and the extracellular matrix are required in the wound healing process. The normal phase of wound healing involves hemostasis, inflammation, proliferation and remodeling. Each phase is distinct, continuous and overlapping 1. The initial responses in tissue injury are wound clearing, vasoconstriction followed by vasodilatation and platelet aggregation. The inflammatory phase is marked by erythema, swelling, warmth and pain 2. In the late inflammation phase, macrophages, neutrophils and lymphocytes migrate to the wound area and release cytokines such as tumor necrosis factor (TNF), transforming growth factor (TGF) and interleukin (IL). These cytokines will stimulate cell migration and proliferation and formation of the tissue matrix 3. The proliferative phase is characterized by the formation of granulation tissue and ephitelization. The final phase in wound healing, tissue remodeling, consists of reorganization of new collagen fibers 2.

There are many types of wounds that can damage skin. Deeper wounds require medical attention to prevent infection and loss of function. However, most wounds are superficial and can be cared for at home. This usually requires cleaning, application of antibacterial ointment and covering with an adhesive bandage. The purpose of medical care for wounds is to prevent infection and loss of function. In several cases, cosmetic treatment is used, but it is not the primary consideration for wound care.

Crude pineapple stem preparations have previously been shown to contain two cysteine proteases: bromelain and ananain 4. Ananain was identified as a minor cysteine protease that possesses distinct substrate and inhibitor binding properties 4. It preferentially hydrolyzes Bz‐Phe‐Val‐Arg‐*p*‐nitroanilide (pNa) substrate. On the other hand, bromelain preferentially hydrolyzes Bz‐Arg‐Arg‐pNa 5. One important pharmaceutical application of cysteine protease is enzymatic debridement of necrotic tissue from ulcers and burn wounds 6.

Tacorin is a crude extract from the stem of *Ananas comosus*, a medicinal plant with highest proteolytic activity in other parts such as fruit and fruit core extract. Tacorin has been developed by Dexa Laboratories of Biomolecular Science (DLBS), one of the biggest pharmaceutical companies in Indonesia, which is exploring various natural compounds for medicinal applications 7, 8, 9, 10, 11, 12, 13. In addition to proteolytic enzyme, tacorin also consists of the amino acids glycine, proline, glutamine and arginine. These amino acids are important in wound healing. To observe the effect of tacorin in wound healing, *in vivo* and *in vitro* studies were conducted. The *in vitro* study used 3T3‐L1 cells, while the *in vivo* study was conducted with rats with an induced injury. The expression of several cytokines and growth factors was quantified with enzyme linked immunosorbent assay (ELISA) using specific antibodies.

## Materials and methods

### Tacorin extraction

Plant parts of *A. comosus* (crown, fruit, fruit stalk/core, butt, leaf and stem) were collected and ground using a blender (Philips, Guangdong, China). The protein fraction from each plant part was extracted using a customized press machine (PT Raja Mesin, Jakarta, Indonesia), followed by separation at 8930 ***g*** at 4 °C for 15 min using a Kubota 7780 centrifuge (Fukuoka, Japan), and filtration through 0.1 μm and 5 kDa membrane with the QuixStand benchstop system (GE Healthcare, Uppsala, Sweden). The filtrate was subsequently dried using a Mini‐Lab fluid bed dryer (Diosna Dierks & Söhne, Osnabruck, Germany). Tacorin was obtained from the plant part of *A. comosus* with highest concentration of protein content and protease activity. Protease in tacorin must be inactivated for cell treatment, and inactivation was carried out by heat exposure at various temperatures (60 and 80 °C) and time incubations (30, 40 and 50 min).

### Assay of tacorin

Characterization of tacorin was carried out by analyzing the protease activity, protein and amino acid contents. The protein profile of tacorin was also obtained by tricine SDS/PAGE.

#### Proteolytic activity assay

The proteolytic activity assay was performed according to Rowan *et al*. 14 with small modifications. A volume of 1.25 mL of the reaction mixture containing 0.65% casein (Sigma‐Aldrich, St Louis, MO, USA) in 20 mm potassium phosphate buffer (pH 7.5) was added to a 0.25 mL sample. The mixture was incubated for 10 min at 37 °C. The reaction was stopped by adding 250 μL of 110 mm trichloroacetic acid (Sigma‐Aldrich). A blank was prepared by adding trichloroacetic acid to the crude enzyme, followed by the substrate. After vortexing for 5 s, 2 mL of reaction mixture was placed in an Eppendorf tube and centrifuged at 9200 ***g*** for 10 min. A half‐volume of supernatant was added to 1.25 mL Na_2_CO_3_ (Sigma‐Aldrich) and 0.25 mL Folin (Merck, Darmstadt, Germany) reagent. The absorbance was measured by UV/Vis spectroscopy at 660 nm. One unit of tacorin is the amount of enzyme that hydrolyzes casein to produce color equivalent to 0.5 nmol of tyrosine per minute at 37 °C and pH 7.5 (colored with Folin‐Ciocalteau reagent, Darmstadt, Germany).

#### Protein content assay

Protein concentration was quantified using the Bradford method 15. One hundred microliters of sample was added to 2 mL of Bradford reagent [100 mg Coomassie G250 (Sigma‐Aldrich), 50 mL of 95% ethanol (Sigma‐Aldrich), 100 mL of phosphoric acid 85% (Sigma‐Aldrich), and water to 1 L]. The mixture was incubated for 10 min at room temperature. The absorbance was read at 595 nm. Bovine serum albumin fraction V (Merck) was used as a reference standard.

#### Protein profile assay

The protein profile of tacorin was performed by tricine SDS/PAGE in 10% and 16% gel concentration using ultra low molecular mass markers (1.02–26.6 kDa). Proteins were visualized by Coomassie Brilliant Blue R‐250 16.

#### Amino acid profile assay

The amino acid profile was analyzed using high performance liquid chromatography (HPLC). Tacorin was injected into a Waters AccQ‐Tag amino acid analysis column (3.9 × 150 mm; Waters Corp., Milford, MA, USA) and eluted with a mixture of AccQ‐Tag eluent A, acetonitrile and HPLC‐grade water, based on the manufacturer's instructions.

### *In vivo* study of tacorin

#### Animals

A total of 14 female Wistar rats (*Rattus novergicus*), weight 170–220 g, were used in this study, seven rats used for the negative control group and the rest for the tacorin group. Rats were caged individually in polysulfone cages and housed under standard conditions (18–25 °C, relative humidity < 70%, 12 h light/dark cycle). All procedures in this study have been reviewed and approved by Dexa Laboratories of Biomolecular Sciences Animal Care and Use Committee with protocol number DOC‐DLBS‐PROC‐APC‐027 and carried out in accordance with Association for Assessment and Accreditation of Laboratory Animal Care International (AAALAC International).

#### Animal treatment

After 1 week of acclimatization, the rats were anesthetized with ketamine (75 mg·kg^−1^ bw) and xylazine (10 mg·kg^−1^ bw) intraperitoneally prior to wound induction. The abdomen was opened by making a vertical incision. A 2.5 cm midline abdominal excision was made from the midpoint of the abdomen to the anterior of the urethra, and then surgery was performed for 2 cm on each side right and left of the uterine area. The wound was then closed by the one‐layer closure technique with continuous lock stiches of 4.0 chromic catgut sutures.

#### Effect of tacorin on wound healing

Wounded rats were divided into an untreated group (negative control group, purified water 1 mL·kg^−1^ bw) and a treated group (tacorin 80 mg·kg^−1^ bw – human dose). Purified water and tacorin were daily administered by the oral route. Then a 1.7% (v/w) blood sample of each rat was taken daily up to 5 days (D0–D5). Blood samples were further analyzed for the expression of TNF‐α and TGF‐β. Tissue was collected from the left and right of the rats' uterine area at days 0, 3, 5 and 7 and were used for analysis of expression of matrix metalloproteinase 2 (MMP‐2). Table 1 shows the design of the animal study.

**Table 1:** Experimental design of animal study

	Observation								
Day	−7 to −1	0	1	2	3	4	5	6	7
Treatment	Acclimatization	Uterine incision			Uterine incision		Uterine incision		Uterine incision
		Blood sampling	Blood sampling	Blood sampling	Blood sampling	Blood sampling	Blood sampling		
		Euthanasia group day 0	Euthanasia group day 1	Euthanasia group day 2	Euthanasia group day 3	Euthanasia group day 4	Euthanasia group day 5		Euthanasia group day 7

### *In vitro* study of tacorin

#### Cell line and treatment

The 3T3‐L1 cell line (American Type Culture Collection CL‐173, Rockville, MD, USA) was maintained in DMEM supplemented with 10% BSA and 1% penicillin/streptomycin (Life Technologies, Carlsbad, CA, USA). Cells were seeded at a density of 4 × 10^3^ cells per well in 96‐well plates and incubated in complete medium for 24 h before use. Then, cells were incubated in serum‐free medium for another 24 h to completely diminish serum in the medium. Treatment was conducted with heat‐inactivated tacorin and bromelain (Sigma‐Aldrich) as a comparator. Cells were treated at various concentrations of both proteins for 24 h. Cell viability was quantified with a 3‐(4,5‐dimethylthiazol‐2‐yl)‐2,5‐diphenyltetrazolium bromide tetrazolium (Promega, Madison, WI, USA) assay.

#### Enzyme linked immunosorbent assay

The expression levels of TNF‐α, IL‐6 and TGF‐β were measured with ELISA using specific antibody (Santa Cruz Biotechnology, Dallas, TX, USA; Abcam, Cambridge, MA, USA). One hundred microliters of diluted antigen (rat serum, 2 μg·μL^−1^ in phosphate‐buffered saline; PBS) was immobilized in a 96‐well microtiter plate (Nunc, Rockilde, Denmark) overnight at 4 °C. After incubation, the plates were washed three times in washing buffer (twice with PBS, once with Tris‐buffered saline and Polysorbate 20). One hundred microliters of blocking solution [5% skim milk (Sigma‐Aldrich) in PBS] was added to each well. Afterwards, 100 μL of primary antibody (1 : 1000; Santa Cruz Biotechnology, Abcam) in blocking solution was added, followed by incubation for 2 h at room temperature on a shaker. Plates were washed as before. One hundred microliters of secondary antibody‐tagged horseradish peroxidase (1 : 1000; Santa‐Cruz Biotechnology) in blocking solution was added to each well. The plates were incubated for 1 h at room temperature. Finally, the plates were washed four times and 50 μL of tetramethylbenzidine (Sigma‐Aldrich) was added. The color intensity of the solution was read in a microtiter reader at 650 nm.

#### Zymogram analysis

The expression of MMP‐2 was detected using zymogram analysis. Approximately 25 mg of uterine tissue was extracted with 1 mL of isolation buffer (1% Triton X‐100, 0.5 m Tris/HCl pH 7.6, 0.2 m NaCl and 10 mm CaCl_2_) for 30 min. The suspension was frozen and thawed twice in a deep freezer, and then centrifuged at 2500 ***g*** for 30 min. The supernatant was collected and dialyzed on ice‐cold dialysis buffer (50 mm tris/HCl pH 7.6, 0.2 m NaCl and 5 mm CaCl_2_) for 48 h. The extracted protein was then diluted in 10 μL of zymogram buffer (0.25 g SDS, 0.3125 mL of Tris base pH 6.8, 0.5 mL of bromphenol 1%, 0.5 mL of glycerol and 1.18 mL of purified water) and analyzed by electrophoresis (SDS/PAGE) using 10% acrylamide. After electrophoresis, the electrophoretic gel was incubated in 2.5% (v/v) Tween 20 for 30 min, in 50 mm potassium phosphate buffer (pH 7.0) for 3 h, and then stained with Coommassie Brilliant Blue R‐250 for 30 min and destained using destaining solution (purified water, ethanol and glacial acetic acid 8 : 1 : 1 v/v/v).

## Results and Discussion

### Characterization of tacorin

**Figure 1:**
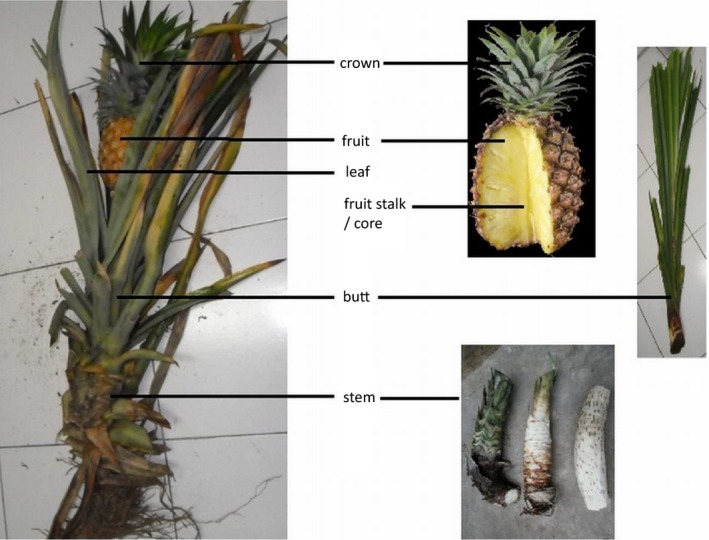
Macroscopic visualization of *Ananas comosus* plant from crown to stem.

*Ananas comosus* was obtained from a supplier from Subang, West Java, Indonesia and identified by Research Center for Biology, Indonesia Institute of Science, Bogor, Indonesia (Fig. 1). Water extract of pineapple contains a number of proteolytic enzymes 17, 18. Protein concentrations from various parts of pineapple extracts were quantified and the highest protein concentration was obtained from stem (Table 2). For further experiments, we used extract from pineapple stem, referred to as tacorin.

**Table 2:** Protein concentration of various parts of pineapple extract

Part of pineapple	Protein content (mg·g^−1^)	Protease activity (U·g^−1^)	Specific activity (U·mg^−1^ protein)
Crown	5.4	235.0	43.3
Fruit	9.9	3298.6	333.2
Fruit stalk/core	4.3	786.5	182.9
Butt	1.9	0.0	0.0
Leaf	10.0	0.0	0.0
Stem	7.1	22 319.8	3143.6

**Figure 2:**
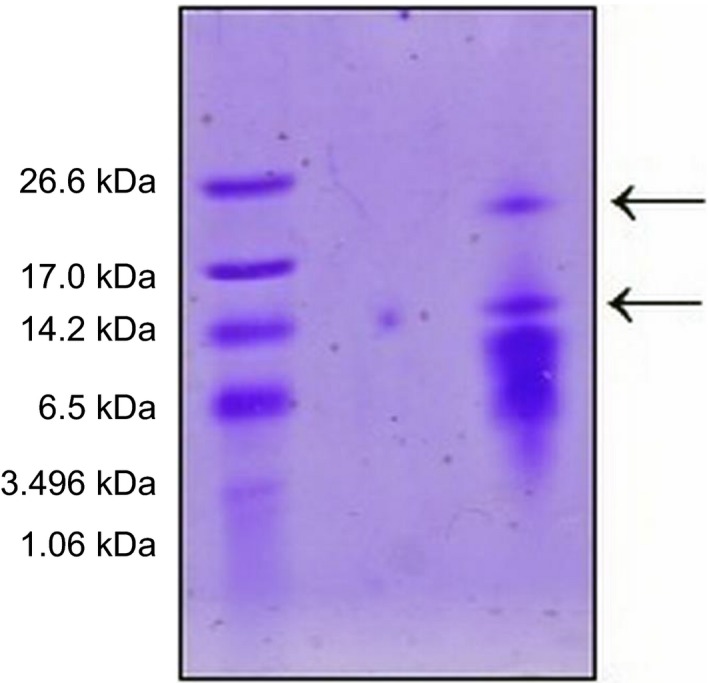
Profile of major proteins of tacorin determined by SDS/PAGE. Lane 1, ultra low molecular mass protein standard; lane 2, tacorin.

The protein profile of tacorin was analyzed using SDS/PAGE and there were found to be two types of protein with molecular masses around 15 and 25 kDa (Fig. 2). These results were similar to ananain from *A. comosus* reported by Rowan *et al*. 4. Another report, from US Patent 7833963, described various proteins with masses of 15.07, 25.85 and 27.45 kDa as being present in bromelain derived from *A. comosus*.

**Figure 3:**
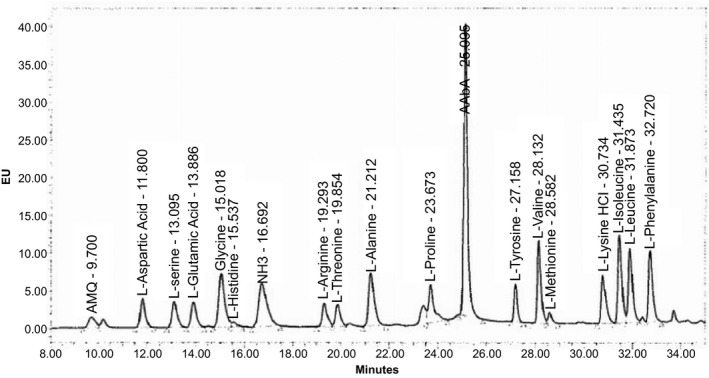
Amino acid profile of tacorin by HPLC using the Waters AccQ‐Tag amino acid analysis column (3.9 × 150 mm) as stationary phase, and a mixture of AccQ‐Tag eluent A, acetonitrile and HPLC‐grade water as mobile phase.

It is well understood that sufficient protein is required in the wound healing process due to the increased need of protein for tissue regeneration and repair. Specific amino acids such as arginine and glutamine have been detected as important constituents in wound healing. Arginine is a non‐essential amino acid that is important in protein synthesis 18, 19. Adequate arginine in tissue appears to be an essential parameter for efficient wound repair. Glutamine is used by inflammatory cells within the wound for proliferation and as a source of energy. Fibroblast cells use glutamine for similar purposes, as well as for protein and nucleic acid synthesis 20, 21. An analysis of amino acids was conducted using HPLC with the profile shown in Fig. 3. Tacorin contains 6.07% total amino acids. Figure 3 and Table 3 show that glycine, proline, glutamine, alanine and arginine are major amino acids in tacorin. Since arginine and glutamine are important in the wound healing process, this might explain the mechanism of tacorin as a wound healing accelerator.

**Table 3:** Amino acid composition of tacorin by HPLC. n.d., not detected: limit to detection of lysine, 455.81 p.p.m.; limit to detection of cysteine 938.38 p.p.m

Amino acid	Amino acid concentration (%)
Essential amino acids	
l‐Arginine	0.5
l‐Histidine	0.08
l‐Isoleucine	0.41
l‐Leucine	0.33
l‐Lysine	n.d.
l‐Methionine	0.09
l‐Phenylalanine	0.3
l‐Threonine	0.31
l‐Valine	0.44
Non‐essential amino acids	
l‐Alanine	0.65
l‐Cysteine	n.d.
l‐Glutamine	0.71
l‐Glycine	0.91
l‐Proline	0.89
l‐Serine	0.45

### The activity of tacorin

*In vivo* study has revealed that the recovery progress of a wound in a group of rats treated with tacorin was faster than in an untreated group, indicated by the decrease of wound area and degree of uterine wound healing. These data have been reported in our previous study by Nailufar *et al*. 22. They demonstrated that after 3 days of treatment, the degree of wound healing in the tacorin group increased significantly, and the degree of uterine wound healing at day 7 reached up to 90% compared with the untreated group.

This study described the mechanism of action of tacorin in the wound healing process. However, the mechanism of action of bromelain has been reviewed. Bromelain has been reported to have anti‐inflammatory properties, which are mediated through the following factors: increased serum fibrinolytic activity, reduced plasma fibrinogen levels, decreased bradykinin levels, decreased prostaglandin E_2_ and thromboxane A2, which in turn decreased prostaglandin levels. Another *in vitro* study reported that bromelain treatment activates natural killer cells and increases the production of TNF‐α, interferon‐γ, IL‐1, IL‐2 and IL‐6. Bromelain was also demonstrated to induce cytokine production in human peripheral blood mononuclear cells, which leads to the production of TNF‐α, IL‐1β and IL‐6 in a time‐ and dose‐dependent manner 23, 24, 25, 26, 27, 28, 29, 30.

**Figure 4:**
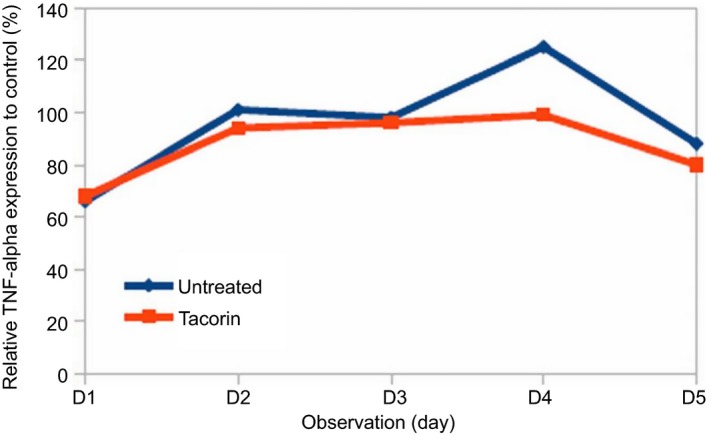
Inhibition of TNF‐α expression in rat serum treated with tacorin compared with untreated group, analyzed using ELISA.

Our study demonstrated that TNF‐α expression decreased when the animal was treated with tacorin (Fig. 4). A pronounced decrease was observed on day 5 (Fig. 4). This result suggested that tacorin protects the progression of inflammation via suppression of TNF‐α level. TNF‐α is a pleiotropic inflammatory cytokine produced by several types of cells, especially by macrophages. TNF‐α is an acute phase protein that initiates a cascade of cytokines and increases vascular permeability, thereby recruiting macrophages and neutrophils to a site of infection 31.

**Figure 5:**
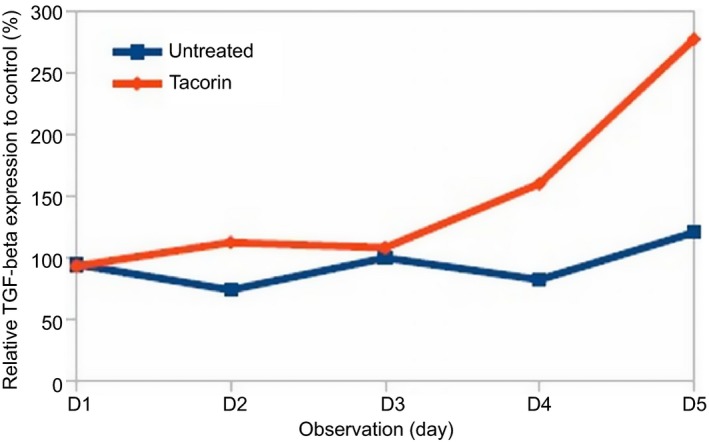
Increased expression of TGF‐β after treatment with tacorin compared with untreated group, measured with ELISA.

In contrast to TNF‐α level reduction, treatment with tacorin enhanced TGF‐β expression (Fig. 5). TGF‐β is a multifunctional protein that controls proliferation, differentiation and other functions in many cell types. This protein interacts with a conserved family of cell surface serine/threonine‐specific protein kinase receptors and generates intracellular signals 32.

**Figure 6:**
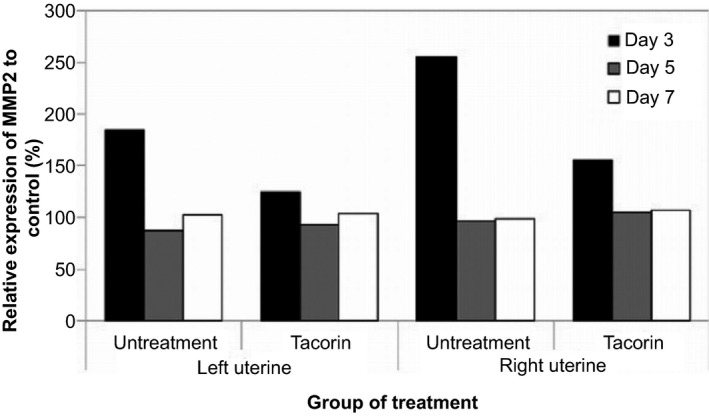
MMP‐2 expression by zymograph analysis from injured rat treated with tacorin or untreated after incubation for 3, 5 and 7 days.

In addition to TNF‐α and TGF‐β, we also quantified the expression of MMP‐2 protein from the treated and untreated groups. At the third day after treatment, a high expression of MMP‐2 was detected in untreated rat, both in left and right uterine areas. Thus, the expression of MMP‐2 was reduced at days 5 and 7. However, in rats treated with tacorin, the level of MMP‐2 was not significantly different at days 3, 5 and 7, which indicates that the recovery process of the treated group was faster than that of the untreated group (Fig. 6). The expression of pro‐MMP‐2 remained constant.

Matrix metalloproteinases (MMPs) are a cell‐derived proteolytic enzyme family with 26 identified members 33. MMPs function in the breakdown of extracellular matrix in normal physiological processes, such as embryonic development, reproduction and tissue remodeling, as well as in disease processes, such as arthritis and metastasis 6. Most MMPs are secreted as an inactive pro‐protein that is activated when cleaved by extracellular proteinase. The protein cleavage activity of MMPs is balanced in time and spatially by cell‐secreted inhibitors called tissue inhibitors of metalloproteinases 34.

Tacorin is a water extract containing proteolytic activity even toward 3T3 cell surface protein that contributes to cell adhesion. Protease‐treated cells had reduced contact with neighboring cells and the plate surface caused by digestion of the cell surface proteins that are involved in cell adhesion. The proteolytic digestion of membrane proteins by protease also showed complete inhibition of fibroblast adhesion on fibronectin‐coated plastic dishes. Therefore, the proteolytic enzyme in tacorin must be inactivated prior to use to avoid false‐negative result. This inactivation was conducted through a physical (heat) method. The proteolytic activity assay of tacorin was conducted using casein 35.

The influence of temperature and incubation time on bromelain activity was reported in several studies 3, 35, 36, 37. Commercial bromelain from pineapple stems was reported to be completely inactivated by heating for 30 min at 60 °C. Bromelain from frozen pineapple fruit of *Bromelia balansae* Mez had no activity when exposed at 37 °C for 120 min. Complete activity loss of *B. balansae* Mez is observed when incubated at 75 °C. The activity of bromelain from *A. comosus* was decreased by 20% when incubated at 50 °C and 100% when incubation was continued at 80 °C for 8 min.

**Figure 7:**
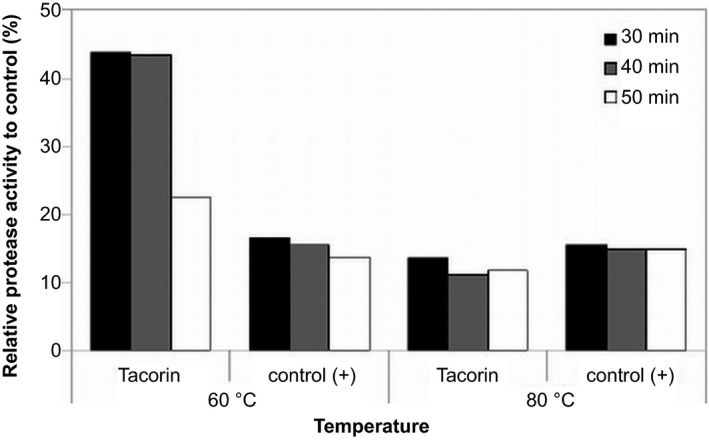
The influence of temperature on protease activity of tacorin and protein control at different incubation times.

**Figure 8:**
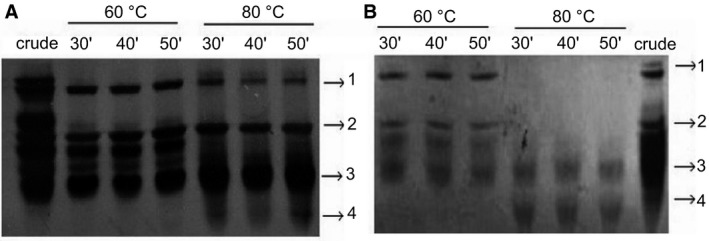
Silver stain analysis of (A) protein control and (B) tacorin.

We examined the effect of temperature on tacorin proteolytic activity. Bromelain was also studied as a control. Upon exposure at 60 °C for 30 min, the activity of tacorin was reduced by 60% (Fig. 7). For longer exposure time, the protease activity of tacorin was reduced by 75%. The activity of control protein remained at a level of 15% in the same condition. Silver‐staining resulted in bands (Fig. 8, bands 2 and 3) that remained even after exposure at 60 °C for 50 min. However, bands 1 and 2 decreased at 80 °C exposure. A new band, suggested to be protein degradation product, appeared after both samples were exposed at 80 °C (Fig. 8, band 4). Thus, inactivation of tacorin and control protein was performed by heating at 80 °C for 30 min.

**Figure 9:**
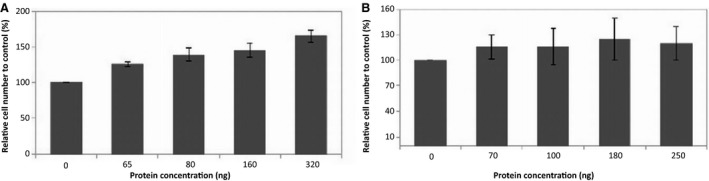
Effect of heat inactivation on (A) tacorin and (B) protein control on 3T3‐L1 cell viability.

Several parameters relevant to the wound healing process are cell regeneration, including proliferation, cell growth and maturation. To observe the effect of tacorin on the proliferation phase, we measured the viability of 3T3 cells in the presence and absence of tacorin and control protein. Tacorin treatment increased cell viability in a dose‐dependent manner (Fig. 9). At the highest concentration of tacorin, the number of cells was nearly double as compared with control. Similarly, when the cells were treated with protein control, the number of cells was 1.5 times higher than control. The ability of tacorin in inducing wound healing is suggested through its ability to promote fibroblast cell growth. Fibroblast proliferation is important in the formation of granulation tissue that is needed for wound closure. Fibroblast growth is related to neo‐angiogenesis and extracellular matrix secretion that needed for cell ingrowth and tissue development, and the production of some cytokines and growth factors 38, 39, 40, 41.

## Conclusion

Tacorin is a crude protein extracted from *A. comosus* stem. The bioactive protein fraction in tacorin contains glycine, proline, glutamine and alanine, which are amino acids that are important in the wound healing process. The effects of protein and amino acid in the wound healing process are through remodeling the expression of cytokines and growth factors. In addition, those proteins and amino acids are involved in inflammation and tissue regeneration as well. Thus, tacorin is a promising wound healing therapeutic agent.

## Author contributions

PR designed the experiments, prepared the paper, provided and characterized tacorin, performed the *in vivo* study and carried out data analysis. LA contributed to writing the paper, planned and performed the *in vitro* study and analyzed data. RRT participated in supervising the whole experiment, and reviewed the entire data and the manuscript.

## Conflict of interest

The authors are responsible for the content and writing of the paper and declare no conflict of interest in this work. The corresponding author is fully responsible for the submission of this publication.
